# Unveiling Ocular Manifestations in Systemic Lupus Erythematosus

**DOI:** 10.3390/jcm13041047

**Published:** 2024-02-12

**Authors:** Mutali Musa, Ekele Chukwuyem, Oluwasola Michael Ojo, Efioshiomoshi Kings Topah, Leopoldo Spadea, Carlo Salati, Caterina Gagliano, Marco Zeppieri

**Affiliations:** 1 Department of Optometry, University of Benin, Benin City 300238, Nigeria; mutali.musa@uniben.edu; 2 Centre for Sight Africa, Onitsha 434112, Nigeria; 3 School of Optometry and Vision Sciences, College of Health Sciences, University of Ilorin, Ilorin 240003, Nigeria; 4 Department of Optometry, Faculty of Allied Health Sciences, College of Health Sciences, Bayero University, Kano 700006, Nigeria; 5 Eye Clinic, Policlinico Umberto I, “Sapienza” University of Rome, 00142 Rome, Italy; 6 Department of Ophthalmology, University Hospital of Udine, 33100 Udine, Italy; 7 Faculty of Medicine and Surgery, University of Enna “Kore”, Piazza Dell’Università, 94100 Enna, Italy; 8 Eye Clinic, Catania University San Marco Hospital, Viale Carlo Azeglio Ciampi, 95121 Catania, Italy

**Keywords:** systemic, lupus erythematosus, autoimmune, corticosteroids

## Abstract

Systemic Lupus Erythematosus (SLE) is a complex autoimmune disorder characterized by immune dysregulation and multi-organ involvement. In this concise brief review, we highlight key insights into Ocular Systemic Lupus Erythematosus (SLE), an intricate autoimmune disorder with diverse organ involvement. Emphasizing the formation of autoantibodies and immune complex deposition, we delve into the inflammation and damage affecting ocular structures. Clinical presentations, ranging from mild dry eye syndrome to severe conditions like retinal vasculitis, necessitate a comprehensive diagnostic approach, including clinical exams, serological testing, and imaging studies. Differential diagnosis involves distinguishing SLE-related ocular manifestations from other autoimmune and non-inflammatory ocular conditions. The multidisciplinary management approach, involving rheumatologists, ophthalmologists, and immunologists, tailors treatment based on ocular involvement severity, encompassing corticosteroids, immunosuppressive agents, and biologics. Follow-up is crucial for monitoring disease progression and treatment response. Future perspectives revolve around advancing molecular understanding, refining diagnostic tools, and exploring targeted therapies. Novel research areas include genetic factors, microbiome composition, and biotechnology for tailored and effective SLE ocular treatments.

## 1. Introduction

Systemic lupus erythematosus (SLE) is a chronic autoimmune systemic disorder of the connective tissue that does not affect only one certain organ but has a diversified effect all over the body [1,2]. The etiology of SLE is may be idiopathic, genetic, hormonal, or environmental [3]. Choudhary et al. have suggested that SLE is nine times more common in females as compared to males [4]. SLE may either be of juvenile-onset or adult-onset [5]. In its early stages, both hereditary and acquired immune systems are implicated, leading to the activation of T and B cells. The result is an overproduction of pro-inflammatory cytokines [3]. The diagnosis of SLE is difficult and requires a series of tests and findings which can be systemic or ocular. Approximately one-third of those diagnosed with SLE experience ocular manifestations that range from mild to severe sight-threatening conditions. Ocular manifestations of SLE include keratoconjunctivitis sicca, uveitis, and posterior segment pathologic signs [6]. It has been found that symptoms of SLE in the eye, such as pathological changes in the posterior segment, and other symptoms that may be observed systemically aid in diagnosis as well as treatment for SLE [7].

The involvement of the orbital tissue in SLE is rare and includes the inflammation of the orbit, myositis (infarction of orbital muscles), exophthalmos, panniculitis, subcutaneous orbital tissue inflammation, and orbital edema. Common symptoms include ophthalmoplegia, reduced vision, ocular proptosis, eyelid lesions, and orbital inflammation [1,8]. Elevated levels of creatine kinase, aldolase, CRP, procalcitonin, and myoglobins are reported from laboratory test results [1,2,3,4,5,6,7,8,9].

Dry eye syndrome is commonplace in SLE [10]. Dry eye associated with SLE may be caused by abnormalities of the lacrimal gland, lacrimal duct, conjunctiva, cornea, and meibomian glands, and encompasses both aqueous-deficient and evaporative dry eye [11]. Patients with SLE have been found to present with severe dry eye symptoms, which might enable the diagnosis of dry eye as an indicator for diagnosing SLE [12]. Reduced corneal sensitivity is a hallmark of severe dry eye that may alter the patient’s perception of ocular discomfort symptoms [13]. Reduced ocular sensation may offer insufficient feedback to the central nervous system via the ophthalmic nerve, leading to less efferent stimulation to the lacrimal gland, decreased tear production, and the encouragement of a vicious cycle [14].

Scaly, pigmented lesions and a cheek rash of the skin of the eyelids and adnexa have been found to be associated with SLE. Common signs noticed on the eyelid and skin in SLE are chronic erythema of the eyelids [15]. Also, unilateral and bilateral blepharitis can occur, and lesions of the eyelids appear as lumps or concretions that attach strongly to the overlying skin [16]. A complication of the SLE involvement in the eyelid margin is madarosis, which can be difficult to manage [17]. The accumulation of immunoglobulins in the tissues may result in episcleritis in SLE patients. The classic symptom of this is the dilation of the superficial blood vessels which shrink significantly when phenylephrine is applied topically. The worsening of vision and severe pain in the eyes are not common [18]. Periorbital inflammation together with edema are signs that patients with SLE present with. Circumbulbar swelling is a typical sign of dermatomyositis—approximately 5% of people with SLE present with periorbital heliotropic edema [19].

Secondary to keratoconjunctivitis sicca, ulcerative and infective keratitis may occur [20]. Corneal symptoms, which can emerge as the first or second manifestations of SLE, have a variety of clinical characteristics and may be a pointer to an existing inflammatory process [21,22]. The management of patients with SLE is a multi-specialist approach with rheumatologists, nephrologists, ophthalmologists, optometrists, and other specialists of organs that may be affected due to SLE [23,24].

While existing literature has contributed significantly to our understanding of SLE, this review seeks to make a distinctive and valuable contribution by comprehensively exploring the nuances of SLE. The significance of this review lies in its unique focus on the intricate relationship between SLE and the various structures of the eye, shedding light on aspects that remain less understood. The review serves as a crucial synthesis of current knowledge, aiming to bridge gaps and provide a deeper understanding of the molecular, diagnostic, and therapeutic dimensions specific to ocular manifestations in SLE. The manuscript delves into the underlying molecular mechanisms, diagnostic challenges, and potential treatment strategies, offering a nuanced perspective on Ocular SLE.

## 2. Methodology

**Figure 1:**
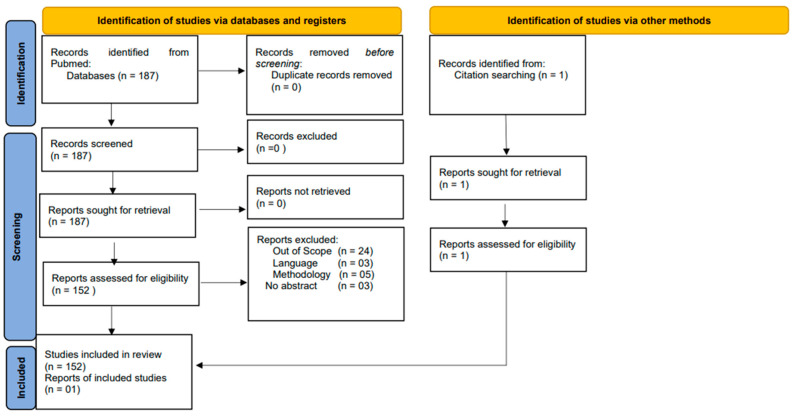
Flow chart according to the PRISMA statement.

The authors conducted a search of the PubMed database using the keywords “ocular systemic lupus erythematosus”. The following search algorithm was subsequently generated by the search engine; ((“ocular”[All Fields] OR “oculars”[All Fields]) AND (“eye”[MeSH Terms] OR “eye”[All Fields]) AND (“lupus erythematosus, systemic”[MeSH Terms] OR (“lupus”[All Fields] AND “erythematosus”[All Fields] AND “systemic”[All Fields]) OR “systemic lupus erythematosus”[All Fields] OR (“systemic”[All Fields] AND “lupus”[All Fields] AND “erythematosus”[All Fields]))) AND (2010:2023[pdat]). A total of 187 records were returned by the search string. Two authors checked each record for language, relevance to the topic, and content. A PRISMA [25] table is used to show the selection process below. Figure 1 shows the flow chart according to the PRISMA statement.

Articles were also reviewed by authors and classified according to the type of articles. Initial categorization of the 187 results revealed 73 case reports, 1 clinical trial, 57 original articles, and 43 reviews. After sorting according to search criteria, a total of 152 articles were included comprising 69 case reports, 1 clinical trial, 50 original articles, and 32 reviews.

## 3. Multidisciplinary Diagnosis

Ocular manifestations seen in lupus are usually not specific to the disease and can be found in other autoimmune-related diseases such as rheumatoid arthritis, reactive arthritis, primary Sjogren’s syndrome, multiple sclerosis, neuromyelitis optica, etc. [26,27,28,29,30]. In fact, systemic conditions like collagen vascular disorders are also implicated in the pathogenesis of SLE [31]. Sitaula et al. however suggested that about 47% of SLE patients express ocular complications [32]. Retinal manifestations in SLE may mimic those found in vasculopathy [33,34,35], trauma, choriodopathy [36], metabolic diseases such as diabetes, and infections. The widespread, nonspecific nature of ocular as well as systemic manifestations of systemic lupus erythematosus heralds the need for a systematic approach towards its diagnosis [37].

Thus, the identification of the nature of the disease requires a comprehensive evaluation, including clinical exams, serological testing, and imaging studies like fluorescein angiography, optical coherence tomography, and adaptive optics imaging [38,39,40]. Serologic evaluation and expressed signs and symptoms make up the mainstay in identifying, as well as differentiating systemic lupus erythematosus from other disease processes with similar physical presentation and serologic expression [41]. Other evaluative procedures such as ophthalmic and multi-organ imaging procedures are employed in detailing expressed signs and symptoms as well as establishing the degree of involvement of these tissues [42,43]. Moreover, ophthalmic investigative procedures such as OCT, and angiography may aid in the early detection of micro-vascular abnormalities and other related pathological findings thus, aiding the early initiation of SLE-specific diagnostic tests [44,45,46,47,48]. Serologic evaluation of anti-nuclear antibodies (ANAs) and anti-extractable nuclear antigens (Anti-ENAs) forms the bedrock in identifying systemic lupus erythematosus and can be ruled out in the absence of a positive result [49,50]. There are various available methods employed for accessing serum anti-nuclear antibodies and anti-extractable nuclear antigens as well as arriving at a clinical conclusion; the immunofluorescent anti-nuclear antibody test has remained for many years the gold standard in the diagnosis of this disease [51]. Enzyme-linked immunosorbent assay (ELISA) and indirect immunofluorescence are commonly used, and the pattern of fluorescence in indirect immunofluorescence can imply the kind of serum antibody. Although ANA is positive in almost all cases of systemic lupus erythematosus, a positive ANA test result does not necessarily imply the presence of systemic lupus erythematosus and should best be interpreted in the appropriate clinical context, since ANA is associated with malignancies, infections, and other autoimmune diseases [52]. The sensitivity and specificity of ANA decreases with the increased prevalence of multiple medical disorders. However, ANA screening along with anti-dsDNA titer is highly sensitive and specific for systemic lupus erythematosus [53]. Although anti-smith antibody (anti-sm antibody) may possess limited sensitivity to systemic lupus, it is highly specific for the disease and has been suggested to help improve both diagnostic criteria and monitoring [54]. When utilizing the indirect immunofluorescence ANA, titers of 1 > 80 have high enough sensitivity as an entry criterion for systemic lupus erythematosus categorization [55]. Thus, the combination of positive ANA titers with clinical presentations and serologic screening for other ANA subtypes such as anti-dsDNA and anti-sm antibodies could point strongly towards SLE. Increased levels of B-cell activating factor have been implicated in the diagnosis of SLE [56]. Other routine evaluations such as ESR, complete blood count, liver enzyme assay, kidney function, electrolyte panel, and complement component levels can point toward an inflammatory origin and more, detailing the signs and multi-organ involvement [57].

## 4. Diagnostic Criteria

A standardized and operationalized set of guidelines required in characterizing a clinical case as systemic lupus erythematosus was first put forward in 1971 by the American College of Rheumatology (ACR), which was subsequently revised in 1982 and 1997 [58,59,60]. Categorization was based on several criteria such as skin lesions, hematologic disorder, oral ulcers, neurologic disorder, renal disorder, arthritis, etc. [61]. Given the improved understanding of the pathogenesis of systemic lupus erythematosus, criteria definitions were refined, and several characterizing criteria such as low complement component, anti-phospholipid antibody evaluation, and mucocutaneous and neuropsychiatric manifestations were added by the 2012 Systemic Lupus Collaborating Clinics(SLICC) [62]. The SLICC classification criteria proposed that the presence of histology proved nephritis is sufficient to classify SLE if serologic evaluation of ANA or anti-dsDNA yielded positive since SLE is mostly an autoimmune disease requiring at least one immunologic criterion to be present [63]. This improved the sensitivity of the SLICC categorization criteria however, the 1997 ACR criteria had a higher specificity in contrast [64]. Longstanding systemic lupus erythematosus was better classified by existing SLE classification criteria than recent onset disease [65]. Consequently, the need to include new-onset SLE in research studies heralded the 2019 European League Against Rheumatism (EULAR)/American College of Rheumatology classification criteria (ACR) [63]. In order to achieve excellent classification criteria for new-onset SLE, the EULAR/ACR proposed the use of positive ANA titers as a statutory entry criterion, with subsequent additive weighted criteria divided into seven clinical (constitutional, renal hematological, neuropsychiatric, serosal, musculoskeletal, and mucocutaneous) and three immunological (complement proteins, anti-phospholipid antibodies, and SLE-specific antibodies) components [66]. This new system of SLE categorization demonstrated a higher tendency to detect new-onset SLE in research and had a sensitivity of 96.1% and specificity of 93.4% in contrast with 82.8% sensitivity and 93.4% specificity of the 1997 ACR revised criteria and 96.7% sensitivity and 83.7% specificity of the 2012 systemic lupus international collaboration clinic criteria [66].

Differential diagnosis: distinguishing SLE-related ocular manifestations from other autoimmune diseases and inflammatory ocular conditions can be daunting due to the non-specific nature of presentations in SLE [67,68]. Anti-nuclear antibodies, which form the serologic hallmark in SLE diagnosis, are found in numerous diseases as well as in healthy individuals and also aged people. These diseases may include systemic lupus erythematosus, infective conditions, malignancies, rheumatoid arthritis, Sjogren syndrome, scleroderma, psoriatic arthritis polymyositis, primary biliary cirrhosis, drug-induced lupus, autoimmune hepatitis, multiple sclerosis, and juvenile idiopathic [69,70,71]. A range of ocular disorders and diseases such as meibomian gland loss without dry eye symptoms, ocular surface diseases, keratitis, uveitis, retinal capillary abnormalities, neuro retinitis, and choriodopathies are associated with systemic lupus erythematosus [72,73,74,75,76]. Neutrophil extracellular traps are also elevated in many ocular pathologies, including SLE, and are potential targets for management strategies [76]. The nature and severity of ocular involvement can vary and can be easily confused with other great masqueraders such as syphilis, in which case it can carry a significant vision-threatening risk [77]. Accurate differential can be achieved by detailing ocular presentation and eliminating possible masqueraders with SLE-specific antibodies (ANA, anti-dsDNA, and anti-sm antibodies), complement panel levels, and anti-beta 2 glycoprotein 1 antibodies [66].

## 5. Ocular Manifestations of SLE in the Oculovisual System

Systemic lupus erythematosus, subacute lupus erythematosus, and chronic lupus erythematosus, of which discoid lupus is a subset, constitute the various types of the lupus erythematosus spectrum of disease [78]. Clinical presentations are diverse, ranging from mild dry eye syndrome to more severe conditions like retinal vasculitis and optic neuritis [79]. Variable ocular involvement arises from immune-mediated processes affecting various ocular structures, including the conjunctiva, cornea, lacrimal gland, EOMs, sclera, the vascular tonic, and the optic nerve. SLE patients are more likely to visit the eye clinic compared to other patients due to its multipronged symptomatology [80,81]. Yazici et al. reported altered corneal biomechanics in SLE patients as compared to a control group [82]. They advocated that these should be taken into consideration while taking measurements. Zhang et al. also assessed a cohort of 93,345 patients, reporting that corneal hysteresis was positively correlated with SLE [83]. Genetics may also be a confounding factor in antiphospholipid syndrome (APS) in the course of SLE [84,85]. Modrzejewska et al. reported on an isolated case of a neonate with pre-retinal hemorrhage secondary to possible vessel thrombosis, whose mother was a known SLE patient [86].

### 5.1. Anterior Segment

Ocular surface disorders are commonly associated with other chronic inflammatory conditions, more so, keratoconjunctivitis sicca or dry eyes disease constitute the most ocular manifestation in lupus erythematosus [87,88] and severity may correlate with SLE activity [89]. Resch et al. reported a significant increase in corneal Langerhans cells in SLE patients compared to normal patients [90]. Generally, the pathophysiological process of dry eyes disease in chronic inflammatory disease such as SLE involves the cellular infiltration of the sebaceous and serous glands involved in tears production and tears integrity maintenance, and a dysregulation of pro-inflammatory cytokine expression [91,92,93,94]. The pathogenesis of evaporative dry eye in SLE patients entails excessive inflammatory cellular infiltration of the meibomian gland, atrophy, and vascular enrichment around the meibomian gland [95,96]. Keratoconjunctivitis sicca has been known to be characterized by an unbridled production of pro-inflammatory cytokines which eventually leads to incipient or manifest chronic ocular surface inflammatory changes [97,98]. T-helper type 17 lymphocyte (Th-17), which is typified by the production of interleukin-17 (IL-17), a powerful pro-inflammatory mediator, has been specifically identified as the leading cause of chronic ocular surface inflammation and consequent epithelial metaplasia resulting in the loss of goblet cells, the mucin-aqueous adherence complex and subsequent dry eye disease [99,100,101]. A neural origin of excessive cytokine expression by leukocytes due to a failure of the sensory afferent mechanotransduction of mechanosensitive ion channel (Piezo1 and Piezo2) has been proposed as the initial cause of events that could result in the activation of the innate and adaptative immune system, which eventually ends in the dysregulation of autoantigen production and consequent dry eyes disease [3].

### 5.2. Cornea and Conjunctiva

**Figure 2:**
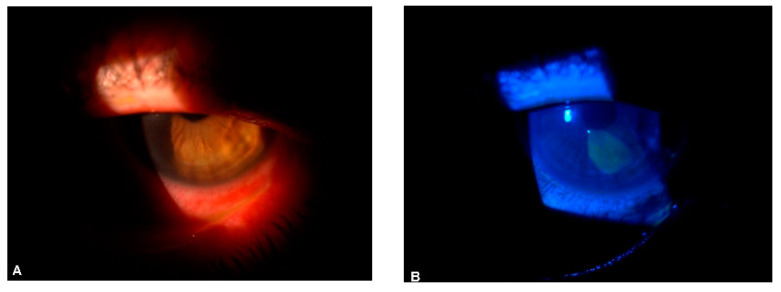
(**A**) SLE-linked corneal epitheliopathy in an adult male; (**B**) Same eye, stained with fluorescein sodium and viewed with a cobalt blue filter.

Cornea involvement in SLE may be heralded by chronic poorly managed or severe keratoconjunctivitis sicca and excessive pro-inflammatory cytokine expression. Hyperosmolar tears in SLE patients [102] may result in high osmotic desiccating stress on the cornea and conjunctival epithelia surface with a subsequent loss of ocular surface epithelia integrity, distorted epithelial metabolism, chronic ocular surface inflammation, epithelial defect (as shown in Figure 2A,B), cornea ulceration, neovascularization, and scarring. Furthermore, the increased apoptosis of ocular surface epithelia cells in SLE [103] may predispose to a higher risk of cornea erosion and keratitis. Recurrent erosive keratopathy, interstitial keratitis [104], kerato-endotheliitis [105], and peripheral ulcerative keratitis [73,106] can manifest in SLE as well as other rheumatologic conditions. Interspecialty management between the ophthalmologist, rheumatologist, and other health care is needed.

### 5.3. Orbit, Extraocular Muscles and Refractive Shift

The involvement of the orbit and extraocular muscles in SLE is rare [89] and is usually a diagnosis of exclusion. The manifestations of orbital involvement, such as orbital apex syndrome [107], panniculitis [108], and orbital vasculitis [109], carry a poor prognosis due to associated optic neuropathy. Clinical features include the limitation of gaze, pain, relative afferent pupillary defect, periorbital edema, conjunctival edema, diplopia, and proptosis [110].

A relative shift of the habitual refractive status in SLE which is termed “acute onset myopia” has been reported, and it is mostly characterized by an increment of the net dioptric power of the eye. The myopic shift in SLE is proposed to result mostly from ciliary spasm secondary to the ciliary body inflammatory response to autoimmune antibodies and freely circulating cytokines [111,112].

Subcutaneous fat inflammation, which is known as panniculitis or lupus erythematosus profundus [113], is a rare ocular finding in discoid lupus erythematosus and is usually associated with enophthalmos due to subcutaneous lipo-atrophic changes and shrinkage [114]. Lupus panniculitis is a refractory disease and early diagnosis is most often difficult and may be confused with panniculitis-like T-cell lymphoma [108].

Orbital vasculitis in SLE results in the inflammation of blood vessels that supply the orbit and the orbital viscera with consequent hypo- or nonperfusion of the orbit and its structures. Orbital ischemic attack due to orbital vasculitis could result in an irreversible loss of vision and neovascular glaucoma secondary to ischemic optic neuropathy and retinal ischemia, respectively [115].

Orbital myositis in SLE is an immune-related inflammatory activity involving the extraocular muscles with a female preponderance [116]. Although rare, bilateral sequential tracheitis has also been reported [117]. Signs and symptoms may include restricted gaze, pain in eye movement diplopia, headache, periorbital edema, and ocular hypertension [116]. It is usually misdiagnosed as orbital cellulitis, as a diagnosis of exclusion a thorough serologic screening, adequate ophthalmic evaluation ultrasound, computerized tomography (CT), and magnetic resonance imaging (MRI) scan are usually required. CT/MRI may demonstrate enlargement and thickening of inflamed extraocular muscles. Treatment is usually with corticosteroid therapy [118].

### 5.4. Uvea and Posterior Segment

The uvea comprises highly vascularized ocular tissues, and its involvement in SLE is rare [119]. Posterior segment manifestations can arise from immuno-complex-induced vasculitis secondary to freely circulating anti-cytoplasmic and anti-nuclear antibodies, hypercoagulable states [120], infection due to treatment-induced immunosuppression, drug toxicities from pharmaceutical agents used in management [121,122,123], and hematological disorders [119]. Posterior segment involvement in SLE includes uveitis [124], occlusive retinal vasculitis, optic nerve edema, combined venous and retinal occlusive disease [125], central retinal artery occlusion [126] macular edema, autoimmune retinopathy [126], retinal and vitreous hemorrhages, exudative retinal detachment, posterior scleritis, optic and retinal neovascular membrane, microangiopathy, tortuous retinal vessels, and perifoveal microvasculature abnormalities [127,128,129].

Lupus vasculitis usually involves small and medium blood vessels, whereas the involvement of larger vessels is mostly rare [130]. Posterior segment involvement resulting from vasculitis carries a poor prognosis [131,132]. The pathophysiology of lupus vasculitis is poorly understood. However, a multiplex interaction involving the complement system, autoantibody, and antigen immune complex; which eventually results in polymorphonuclear leukocytes being unleashed on the vascular endothelium and the recruitment of various immune-related inflammatory moieties has been proposed. This immune-related chronic pro-inflammatory event eventually becomes self-sustained, resulting in the formation of autoantibodies and deposition of immune complexes on the vascular endothelium, in turn leading to inflammation and tissue damage. The binding of autoantibodies to antigens creates a soluble immune complex which is then deposited on the vascular endothelium due to a faulty clearance by the reticuloendothelial system and an increased vascular permeability [130]. This is then followed by a complement fixation cascade which leads to the recruitment of polymorphonuclear leukocytes, complement protein depletion from phagocytosis, and inflammatory reaction [133]. Although rare, the simultaneous presentation of lupus and anti-neutrophilic cytoplasmic antibody (ANCA) has been noted in several instances [134,135,136]. ANCA binds to proteinase3 (PR3) and myeloperoxidase (MPO) antigen resulting in the adherence of neutrophils to the vascular endothelial cells with subsequent inflammation, vascular endothelial damage, and apoptosis [137,138,139].

Furthermore, other auto-antibodies, such as anti-endothelial cell antibody (AECA), have been proposed to induce endothelial cell activation by increasing the expression of endothelial adhesion molecules, such as intracellular adhesion molecule-1 (ICAM-1) and E-selectin, and to alter cytokine production with the consequent secretion of pro-inflammatory cytokines such as interleukin1 [IL1] and tumor necrosis factor [TNF] [140]. This may eventually result in the increased adhesion of leukocytes to the endothelial cell, activation of coagulation, inflammatory endothelial damage, vascular injury, and thrombosis [141]. Menet et al. reported that SLE was implicated in antiphospholipid syndrome with varying ocular morbidities [142,143]. Therefore, freely circulating anti-phospholipid antibodies may bind to the phospholipid fragment of the already injured endothelial cell resulting in various thrombotic cascades and subsequent ophthalmic vaso-occlusive crisis [144].

Retinal and choroidal manifestations in SLE indicate the severity of the systemic condition [145,146]. Retinopathy associated with SLE occurs in approximately 12% of affected individuals, although its incidence is reduced with the use of systemic therapy [146]. Mild retinopathy can be asymptomatic and incidental, severe vascular occlusive retinopathy is characterized by vision impairment, distortions, and visual field abnormalities. Retinal neovascularization, retinal detachment, microaneurysms, artery constriction, lesions at arteriovenous junctions, retinal edema, retinal exudation, and multifocal serous retinal detachment or pigment epithelial detachment are common retinopathies presented in SLE [3].

Central serous chorioretinopathy (CSCR) is a rare ocular manifestation of SLE which may be caused by choroidal ischemia and inflammation. CSCR is characterized by localized serous neuroepithelial detachments within and around the macula. Reported incidences range from approximately 0.004 to 0.007%, with higher prevalence among young and middle-aged men. A total of 60–80% of affected patients reportedly develop unilateral ocular involvement only, while around 30–45% tend to have poor visual prognosis [147].

Intra-ocular infection is a dangerous complication in patients with SLE as a result of immunosuppression. The symptoms of autoimmune diseases are alleviated with methotrexate and azathioprine [148]. Glucocorticoids are contraindicated in central serous chorioretinopathy, however, the use of glucocorticoids in appropriate doses has been used in a patient with SLE associated with renal failure and rapid decrease in vision who had no history of glucocorticoids use, for alleviating both the systemic and ocular manifestation of SLE [147]. A good visual prognosis can be achieved by early diagnosis and aggressive treatment with intravenous cyclophosphamide [119]. Cyclophosphamide and Rituximab have been used aggressively, as reported by other authors, in managing ocular manifestations such as retinal vascular occlusion and occlusive retinal vasculitis [149,150]. Systemic steroids, hydroxychloroquine sulfate (HCQS), blood thinners, immunosuppressants, and laser photocoagulation have been used in managing the posterior complications of SLE [151,152].

An initial symptom of SLE may be optic nerve dysfunction but this usually occurs during the course of the disease. Optic neuritis and ischemic optic neuropathies are implications of the effect of SLE on the optic nerve which results in progressive vision loss and may lead to complete blindness [153]. Abnormal pupil responses, cranial nerve palsies, ocular motility defects, internuclear ophthalmoplegia, transient monocular vision loss, cortical visual impairment, and varying scotomas are less common presentations underlining neuropathies seen in SLE [154]. It should, however, be noted that multiple authors have opined that central visual field testing and microperimetry are not good diagnostic tests for SLE [155]. Optic neuritis in SLE is a result of an ischemic process that leads to subsequent demyelination. Patients’ best-corrected visual acuity correlates to the degree of axon loss. A total of 50% of patients with optic neuritis end up progressing to optic nerve atrophy. The early administration of corticosteroids (intravenous pulsatile steroid therapy), followed with continuously tapered oral doses, and addition of DMARDs, e.g., cyclophosphamide and methotrexate, can mitigate damage [3]. Table 1 summarizes some less common signs and symptoms reported in patients suffering SLE.

## 6. Management and Treatment

Even though the pathogenesis is not completely understood, the etiology of SLE plays a vital role in its management. Systemic lupus erythematosus (SLE) is a chronic diversified autoimmune disease that is widely believed to be influenced by external factors, such as the environment, and intrinsic factors like the genetic makeup of the individual affected, which play a vital role in the development of the disease through several mechanisms [184]. Also, the manifestation of the disease is unique and severity differs among patients [185]. For this reason, mentioned above, the disease presents an important challenge that requires a multidisciplinary approach to its management [186]. Specialists of various medical backgrounds, such as the internist (rheumatologist, nephrologist, endocrinologist, immunologists), neurologist, ophthalmologist, optometrist, dermatologist, and other specialists depending on the patient’s presentation, are needed [187].

In its initial presentation, it is treated with immunosuppressive agents to manage the early signs and symptoms even though this is not curative as patients have to stay on immunosuppressants for a lifetime [74]. In SLE, treatment aims to stave off damage to organs that could be affected as the disease is widespread and affects multiple organs in the body.

The treatment of the disease is dependent on the course of the disease [188]. The outcome of the disease has been significantly improved since the advent of the EULAR guidelines for disease management in 2008. Although this guideline has gone through remarkable reviews i.e., SLEDAI, it stands as the key guide towards excellent management of the condition as the other reviews only strengthened the initial edition [189]. However, other methods of evaluation of the disease are used to measure the disease severity and progression, among them are the following: PGA (Physical Global Assessment), which is a visual equivalent that Physicians use; SLEDAI-2k (Systemic Lupus Erythematosus Disease Activity Index-2000), which is an improvement on SLEDAI; SLAQ (Systemic Lupus Erythematosus Activity Question); LFA-REAL (Lupus Foundation of America Rapid Evaluation of Activity in Lupus); ECLAM (European Consensus Lupus Activity Measurement); and BILAG 2004 index (British Isles Lupus Assessment Group) [190,191]. The pathophysiology of the disease shows remarkable irreversible damage from the inflammation and thus it is important to ascertain this damage through various indicators like the Brief Index of Lupus Damage, SLICC (Systemic Lupus Erythematosus International Collaborating Clinics), and the American College of Rheumatology Damage Index [192].

The above-listed clinical indices/measures are very important in the appropriateness of drugs to be used and their efficacy in the management of the disease. These indicators have a range of values that help clinical decisions on management. For instance, a zero score using SLEDAI shows that there is absolute remission or no active inflammatory activity [193]. However, a score of 1–5 is a pointer to mild disease and inflammatory activity, while a score of 6–10 is a pointer to moderate disease activity in the SLEDAI score [193]. Also, if the score is 3 or greater than it is 3 points to an exacerbation of the disease while a decrease from 3 shows a remission in the activity of the disease [191].

The treatment of the ocular manifestation of SLE is usually targeted at reducing ocular tissue damage with the general treatment focusing on the remission of end-organ damage, improving the outcome of the disease in the long run and improving quality of life [148] In SLE, the treatment is deemed effective if the patient has a remission with an SLEDAI score less than 3. Conventionally, SLE is treated with antimalarial drugs, the commonly used one being hydroxychloroquine [194,195]. Long-term hydroxychloroquine may, however, dispose users to retinopathies [196,197,198]. Martín-Iglesias et al. disputed this, stating that a daily dose of 5 mg/Kg of body weight was safe even after following up on patients after five years [196]. Moschos et al. reported that visual acuity and mfERG scores improved in 42 SLE patients after the cessation of hydroxychloroquine therapy [199]. On the other hand, posterior segment flare-ups have been reported after stopping hydroxychloroquine [200]. Steroidal systemic drugs like prednisolone used in acute phases of the disease are often tapered off and substituted with an immunosuppressant depending on the presentation of the disease [93]. However, these treatment options present diverse side effects on the general condition of the patient on long-term therapy, and effective dose management is recommended to abate its effect [201,202,203,204,205].

The ocular manifestation of SLE affects various ocular tissues. In this review, a systematic approach to the treatment of the involved tissues was performed from the outermost ocular adnexa to the retina (neural layer). Even though orbital tissue involvement is rare, a few studies have shown that there are manifestations in the orbit of patients with SLE where they present with proptosis, enophthalmos, extraocular muscle paresis secondary to orbital vasculitis, chemosis, orbital pain, and orbital myositis, and these are all treated with prescribed systemic immunosuppressant [206]. Also, with respect to orbital tissue involvement, there are cases of discoid lupus erythematosus (DLE) which affects the cutaneous tissues without organ involvement. The treatment for DLE and SLE is with systemic antimalarial therapy and where this fails, systemic immunosuppressants are prescribed. In the treatment of the orbital presentation, corticosteroids are an alternative treatment when there are exacerbations of the orbital inflammatory signs and symptoms and the initial treatments have failed [207].

Documented clinical evidence shows that the lacrimal and the accessory lacrimal glands can be affected in SLE due to mononuclear cellular infiltrates reducing lacrimal fluids produced in the lacrimal glands [208,209]. Hence, keratoconjunctivitis sicca is one of the mild ocular presentations in SLE with a prevalence of one in four patients presenting with it [210]. The treatment for dry eyes in SLE is considered to be best managed with lubricating eye drops while very severe cases may need a short-term or long-term solution like punctal occlusion. In some cases, Tacrolimus ointment (0.03%), a topical immunosuppressant, is prescribed as it reduces the cellular infiltrates from the inflammatory processes [211].

In addition to dry eye disease, SLE has an association with other ocular surface tissues like the cornea as there is sufficient clinical evidence of periodic corneal erosion, corneal peripheral cellular infiltration, keratitis (commonly present as peripheral and interstitial), ulcerative keratitis (less frequent), and rare manifestations like endothelitis [212,213,214]. In the case of the corneal infiltrates, topical steroids are administered with rapid remission [215]. It is known that there is an association between autoimmune diseases like SLE and keratoconus as this gives insight into the pathogenesis and pathophysiology of keratoconnus [216]. LASIK procedures are therefore contraindicated for SLE sufferers [217]. Also, scleritis is a known presentation in SLE and it manifests as both nodular and diffuse scleritis with a known distribution of 1% from other studies [218,219,220]. Necrotizing scleritis is an unlikely manifestation of SLE but where there is the occurrence of such, it often presents with the clinical feature of scleral thinning [219]. Other forms of scleritis like posterior scleritis have been found to be rare but episcleritis has been reported from other studies where the presenting symptoms were mild and self-resolving. Most cases are treated with nonsteroidal anti-inflammatory drugs with good remission [221]. Rarely seen is its manifestation in ocular vascular tissues, but some studies have proven that uveitis from SLE is a result of the inflammation of the adjacent scleral tissues which results in mild cases of uveitis; however, such occurrences of SLE in patients with uveitis are rare in the population [219]. This is found to have a prevalence of 1.38% [26]. Commonly found with uveitis as a sequelae of SLE is that its management with systemic immunosuppressants gives a good remission without hypopyon and reduction in vision [222]. On the other hand, the retina is more affected and a study has shown that 10% of SLE patients have retinal involvement with presentations such as hemorrhages in the retina, tortuosity of the retinal vessels, cotton wool spots, and exudates (hard types), which is indicative of anticardiolipin antibody [223]. Treatment options for retinal complications vary and depend on the severity of the disease, ranging from the use of systemic steroids, to anticoagulant therapy to inhibit the complex cycle between inflammation and thrombosis, and finally, to retinal laser photocoagulation for findings of ischemic retinopathy [93]. Also, neuro-ocular involvement has been seen presenting as papilledema, ischemic optic neuropathy, and optic neuritis with a prevalence of one in every one hundred SLE patients [215].

Longstanding (chronic) treatment with hydroxychloroquine causes nephrotic and ocular complications like toxic maculopathy, extraocular muscle palsy, and cataracts [224]. Obesity is a confounder in patients on hydroxychloroquine [225].

## 7. Future Directions

There are numerous aspects that currently challenge our understanding of this complex autoimmune disorder. SLE’s hallmark immune dysregulation and multi-organ involvement manifest in various ocular structures, including the conjunctiva, cornea, sclera, uvea, retina, and optic nerve. The formation of autoantibodies and immune complex deposition leads to inflammation and tissue damage, presenting with diverse clinical manifestations ranging from mild dry eye syndrome to severe conditions like retinal vasculitis and optic neuritis.

Despite advancements in our understanding, several aspects remain enigmatic. The molecular mechanisms underlying ocular manifestations in SLE are not fully elucidated, prompting the need for further research to unravel the intricacies of this immune-mediated ocular pathology. The role of genetic factors in predisposing individuals to SLE-related ocular involvement is an area requiring in-depth exploration. Understanding the genetic determinants may contribute to identifying individuals at higher risk and developing targeted interventions.

Additionally, the influence of the microbiome composition on the ocular manifestations of SLE remains an intriguing area of investigation. Exploring the interplay between the microbiome and the immune system could offer novel insights into the pathogenesis of ocular complications in SLE, potentially opening avenues for microbiome-targeted therapeutic strategies.

Diagnostic tools for assessing ocular involvement in SLE, while comprehensive, could benefit from refinement. Advanced imaging studies, such as optical coherence tomography, have shown promise, but further developments in non-invasive techniques are warranted to enhance accuracy and early detection. The evolving landscape of biotechnology provides exciting prospects for tailored and effective treatments. Investigating novel biotechnological approaches, such as gene therapies or targeted biologics, holds potential for addressing the specific molecular pathways involved in SLE-related ocular manifestations”.

Future perspectives revolve around advancing our understanding of the molecular mechanisms underlying ocular manifestations, refining diagnostic tools, and exploring targeted therapies. New frontiers in research involve investigating the role of genetic factors, microbiome composition, and novel biotechnological interventions to provide more tailored and effective treatments for ocular involvement in SLE. Killian et al. hinted that anti-IFNα vaccination can potentially prevent morbidity secondary to SLE as it has passed Phase II testing [226].

## 8. Limitations

With regards to the limitations in our review study, it is crucial to explicitly acknowledge the scope and potential biases in the research process. This review primarily focused on literature available through PubMed. While this choice was made to maintain focus and depth within the scope of the paper, it is essential to note that a comprehensive search across multiple databases was not conducted. As a result, there is a possibility that some relevant literature from other databases may not have been included. The selection process involved the consideration of full-text articles. It is essential to acknowledge that grey literature, including conference abstracts and unpublished studies, can contribute valuable insights, and may have limited us from considering data from clinical studies currently underway. Given that SLE is a disease with documented racial disparities, language limitations in the reviewed literature could present a potential impact of language bias on the findings and conclusions of the review.

## 9. Conclusions

Ocular Systemic Lupus Erythematosus (OSLE) represents a complex and multifaceted manifestation of systemic lupus erythematosus (SLE) that significantly impacts the overall well-being of affected individuals. This paper has delved into the various aspects of OSLE, exploring its clinical presentation, diagnostic challenges, and the importance of a multidisciplinary approach in managing this condition. The intricate interplay between immunological dysregulation, genetic factors, and environmental triggers underscores the need for continued research to unravel the underlying mechanisms driving ocular manifestations in SLE. Despite advancements in diagnostic techniques and treatment modalities, challenges persist in accurately diagnosing and effectively managing OSLE. The heterogeneity of ocular manifestations, coupled with the variable course of SLE, necessitates individualized care plans that address both local and systemic aspects of the disease. Collaboration between rheumatologists, ophthalmologists, and other healthcare providers is paramount in achieving optimal outcomes for patients with OSLE. In the treatment of SLE, ocular complications do occur from the systemic treatment options from the corticosteroids, non-steroidal anti-inflammatory drugs, antimalarials, biologic agents, and immunomodulatory agents.

In conclusion, ocular SLE emerges as a complex manifestation of the systemic disease with profound implications for affected individuals. This review has systematically addressed the clinical presentation, diagnostic complexities, and the necessity for a multidisciplinary approach in managing ocular SLE. The intricate interplay of immunological dysregulation, genetic factors, and environmental triggers underscores the ongoing need for research to unravel the mechanisms driving ocular manifestations in SLE. Despite advancements, challenges persist in accurately diagnosing and managing ocular SLE due to its heterogeneity and the variable course of SLE. Tailored care plans, collaboration between healthcare providers, and awareness initiatives are crucial for optimal outcomes. Ongoing research holds promise for uncovering biomarkers and innovative treatments, offering personalized strategies in the era of precision medicine. Continued efforts in raising awareness are essential for early detection, intervention, and improving the overall quality of life for individuals with ocular SLE. In navigating the complexities of ocular SLE, the pursuit of knowledge and targeted therapies remains imperative for enhancing outcomes and the well-being of those affected by this facet of SLE.

## Figures and Tables

**Table 1:** Signs and symptoms reported in patients suffering Systemic Lupus Erythematosus (SLE).

Authors	Signs and Symptoms
Chauhan et al. [156], Budoff and Tsui [157]	Fronsted branch angiitis.
Li et al. [158], Garcia-Soler et al. [159]	Purtscher-like retinopathy
Prakash et al. [160]	Simultaneous occlusion of the central retinal artery and vein, and optic neuritis
Subasi et al. [161]	Changes in macular and peripapillary microvascular density
David et al. [162]	Chorioretinopathy
Alhassan et al. [163], Kuthyar et al. [164], Wu et al. [165]	Retinal vasculitis
Zhang et al. [166]	Unilateral branched retinal occlusion
Liu et al. [167], Shi et al. [168]	Retinal thickness and microvascular alterations
Lin et al. [169], Wang et al. [170]	Scleritis, keratitis, and orbital cellulitis
Braga et al. [171], Agin et al. [172]	Choroidal thickness alterations
Fischer et al. [173]	Child-onset chorioretinopathy
Invernizzi et al. [174]	Drusenoid retinopathy in young adult
Kumar et al. [175]	SLE flare up resulting in retinopathy
Monov et al. [176]	Acute necrotizing scleritis
Park et al. [177]	Acquired enophthalmos.
Jeyachandran et al. [178]	Optic neuropathy
Hu and Peng [179]	Macular infarction
Chin et al. [180]	Polypoidal choroidal vasculopathy
Baglio et al. [181]	Choroidopathy
Salazar et al. [182]	Nine syndromes
Fraga et al. [183]	A cohort of pediatric SLE patients were reported to have significant chloroquine-induced retinopathies and other ocular morbidities

## Data Availability

Data is available in a publicly accessible repository.
